# Substituent Effects in Multivalent Halogen Bonding Complexes: A Combined Theoretical and Crystallographic Study

**DOI:** 10.3390/molecules23010018

**Published:** 2017-12-22

**Authors:** Antonio Bauzá, David Quiñonero, Antonio Frontera

**Affiliations:** Department of Chemistry, Universitat de les Illes Balears, Crta de Valldemossa km 7.5, 07122 Palma de Mallorca (Baleares), Spain; david.quinonero@uib.es

**Keywords:** multivalent halogen bonding interactions, CSD search, RI-MP2 calculations, AIM and NBO analyses

## Abstract

In this manuscript, we combined ab initio calculations (RI-MP2/def2-TZVPD level of theory) and a search in the CSD (Cambridge Structural Database) to analyze the influence of aromatic substitution in charge-assisted multivalent halogen bonding complexes. We used a series of benzene substituted iodine derivatives C_6_H_4_(IF_4_)Y (Y = H, NH_2_, OCH_3_, F, CN, and CF_3_) as Lewis acids and used Cl^−^ as electron rich interacting atoms. We have represented the Hammett’s plot and observed a good regression coefficient (interaction energies vs. Hammett’s σ parameter). Additionally, we demonstrated the direct correlation between the Hammett’s σ parameter and the value of molecular electrostatic potential measured at the I atom on the extension of the C–I bond. Furthermore, we have carried out AIM (atoms in molecules) and NBO (natural bonding orbital) analyses to further describe and characterize the interactions described herein. Finally, we have carried out a search in the CSD (Cambridge Structural Database) and found several X-ray structures where these interactions are present, thus giving reliability to the results derived from the calculations.

## 1. Introduction

Noncovalent forces are considered to be of great importance for the advancement and progress of supramolecular chemistry [1,2]. A deep comprehension of them is vital for chemists working in this discipline, since many chemical and biological phenomena such as molecular recognition, protein-folding, enzymatic reactions, and the formation of supramolecular structures are governed by an intricate combination of noncovalent interactions [3,4,5,6]. Consequently, a proper description and understanding of noncovalent interactions between molecules is important in this field of research. One of the classical and well-known supramolecular forces present in many chemical and biological environments is the hydrogen bonding [7]. In a parallel way, halogen bonding [8] is a noncovalent force that is comparable in strength and directionality features with the hydrogen bonding interaction. Particularly, a halogen bond (R−X···Y−Z, X = halogen, Y = electron rich atom, Z = any atom) has been defined by the International Union of Pure and Applied Chemistry (IUPAC) as the “evidence of a net attractive interaction between an electrophilic region associated with a halogen atom in a molecular entity and a nucleophilic region in another, or the same, molecular entity” [9]. In this context, the ability of halogen atoms to interact with Lewis bases has been known for some time [10]. For instance, Resnati and co-workers have reported the ability of iodo- and bromoperfluoroalkanes to form noncovalent interactions with neutral and charged electron donors, in particular they demonstrated the ability of dihaloperfuoroalkanes to participate as halogen-bond donor moieties in crystal engineering, unveiling the promising potential of halogen bonding interactions in supramolecular chemistry [11,12,13,14,15,16,17,18,19]. Consequently, a series of studies using the CSD were carried out to shed light into the impact of this interaction in solid state chemistry [20,21]. The interest among the scientific community has expanded rapidly due to its importance in biological systems and in the design of new materials. This has led to a plethora of theoretical and experimental studies devoted to this fruitful line of research [22,23,24,25]. Apart from the classical halogen bond, there is a growing interest among the scientific community to understand, in a proper way, the weaker halogen···halogen interactions [26], which have shown a promising future as stabilizing agents of metal complexes [27,28], governing the formation of novel solid state architectures [29,30,31] and even tuning biological and conjugated materials’ properties [32,33,34,35]. In this regard, the presence of both the electron-rich ‘belt’ and the electro-positive σ-hole (Cl, Br and I) are key to describe the two types of X···X interactions, which are usually classified as either type I and II [36].

On the other hand, the analysis and evaluation of the ability of multivalent halogenated compounds to act as Lewis acid centers was firstly analyzed by Landrum and collaborators [37]. They demonstrated that halogen–halogen interactions in these species may be explained as a combination of both donor–acceptor and electrostatic contributions. In other study carried out by Ochiai and coworkers [38], the interactions between trivalent iodine and oxygen for complexation of crown ethers were analyzed. Further examples involve the studies from the groups of Óhair [39] where they analyzed the neighboring-group stabilization of iodine-, arsenic-, and phosphorus-centered oxyanion moieties in deprotonated 2-iodoxybenzoic acid and its analogs, and Wang [40] whose study was devoted to the comparison between monovalent XF and multivalent XF_3_ (X = Br or Cl) halogen bonding complexes. Nowadays, the implications of multivalent halogen bonding interactions in solid state chemistry and more in particular, in the fields of crystal engineering and materials science [41,42,43,44], have opened up new possibilities for supramolecular chemists working in these disciplines.

**Figure 1:**
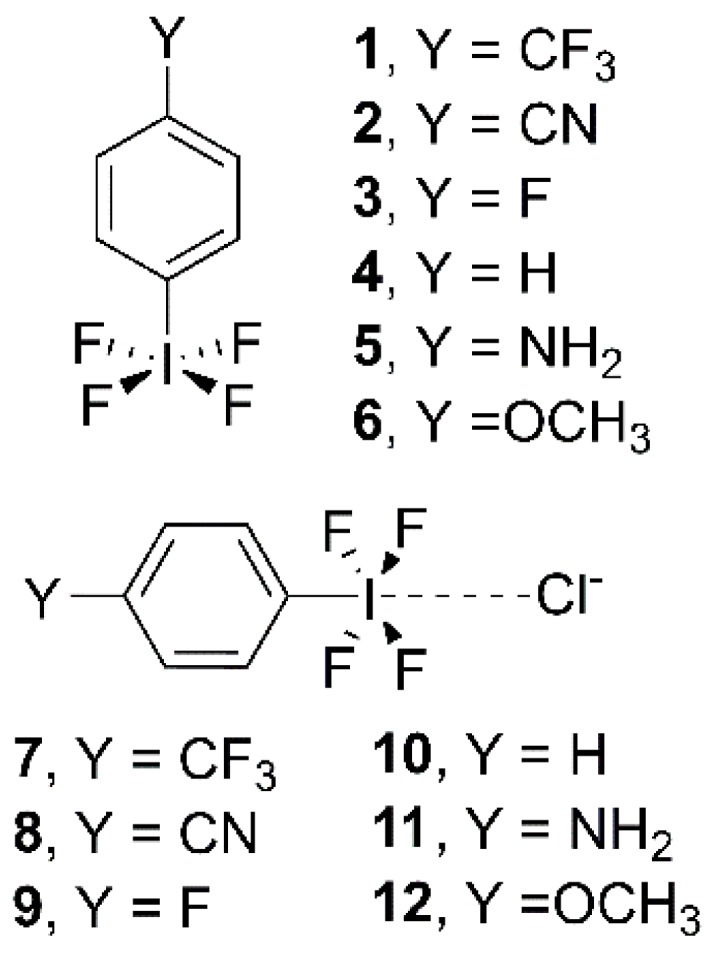
Compounds and Complexes **1**–**12** studied in this work.

In this study, our goal is to analyze the influence of aromatic substitution on the energetic and stability properties of halogen bonding interactions involving multivalent iodine derivatives. For this purpose, we have used C_6_H_4_(IF_4_)Y (Y = CF_3_, CN, F, H, NH_2_, and OCH_3_) as σ-hole bond donors and Cl^−^ as electron rich entity (see Figure 1). We have also obtained the Hammett’s plot in order to correlate interaction energy value (E_BSSE_) and the *p*-substituted σ values. In addition, we have performed a AIM and NBO analyses to further characterize the interactions described herein. Finally, we have performed a search in the CSD in order to find experimental evidence of the impact of multivalent halogen bonding interactions in the solid state.

## 2. Results and Discussion

### 2.1. Preliminary MEP Analysis

**Figure 2:**
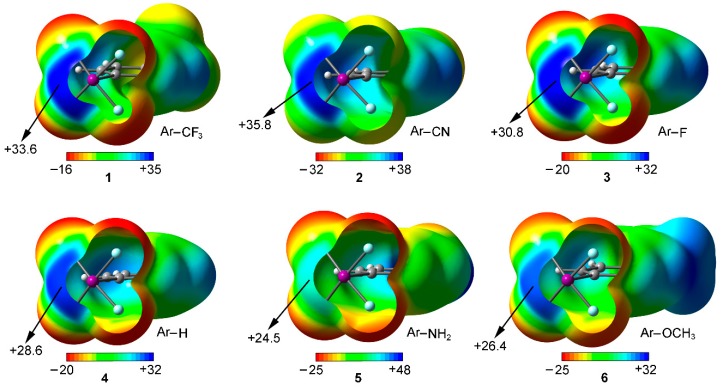
MEP surfaces of Compounds **1** to **6** used in the study. Energies at selected points of the surface (0.001 a.u.) are given in kcal/mol.

We have firstly computed the MEP (molecular electrostatic potential) mapped onto the van der Waals surface for Compounds **1** to **6** (Figure 2). As noted, in all cases a positive electrostatic potential region is found on the extension of the C–X bond, named σ-hole. The presence of this region makes one expect an attractive interaction with an electron rich entity. In addition, the MEP values over the iodine atom are more positive for compounds involving an electron withdrawing substituent (**1** to **3**) than for the rest of the set, showing the following behavior CN > CF_3_ > F > H > OCH_3_ > NH_2_, as known for halogen bonding interactions. Consequently, stronger interaction energy values are expected for complexes involving CF_3_ (**1**) and CN (**2**) substituents than for those involving Compounds **3** to **6**. Finally, it is also worth pointing out that, for Compound **5**, hydrogen bonding interactions involving the NH_2_ group would be more favored than halogen bonds owing to the iodine and hydrogen MEP values (+24.5 and +48 kcal/mol, respectively) however for comparison purposes we will focus our study on the latter interaction.

### 2.2. Energetic and Geometric Results

**Figure 3:**
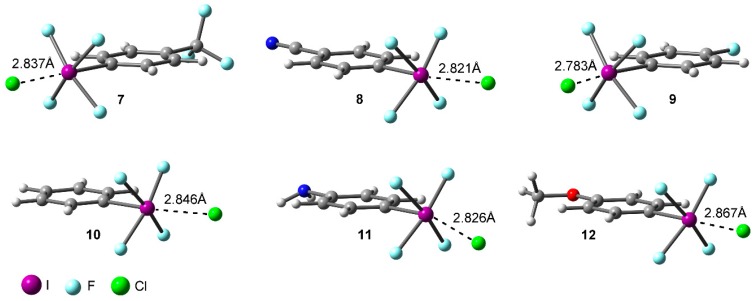
RI-MP2/def2-TZVPD optimized geometries of Complexes **7** to **12**.

Table 1 gathers the interaction energies and equilibrium distances of the optimized Complexes **7** to **12** (see Figure 1 and Figure 3) computed at the RI-MP2/def2-TZVPD level of theory. From the analysis of the results, several points are worth discussing. First, in all cases the interaction energy values are large and negative, ranging between −29.2 and −19.7 kcal/mol. Second, complexes involving electron withdrawing substituents (**7**, **8**, and **9**) obtained more favorable binding energy values than those involving H (**10**), NH_2_ (**11**), and OCH_3_ (**12**), in agreement with the MEP analysis discussed above. Third, Complex **8** involving CN obtained the largest interaction energy value of the study (−29.2 kcal/mol), owing to the strong σ- and π-electron acceptor ability of the CN moiety. On the contrary, Complex **11** involving the NH_2_ group obtained the poorest interaction energy value of the study (−19.7 kcal/mol). Among Complexes **7** and **9** involving CF_3_ and F substituents, the former achieved a more favorable interaction energy value (−27.1 kcal/mol). On the other hand, for Complexes **11** and **12** involving electron donating substituents, slightly different interaction energy values were obtained (−19.7 and −20.8 kcal/mol, respectively) likely due to their similar π-electron donor ability. Finally, Complex **10** (H) obtained an interaction energy value in between Complexes **9** (F) and **12** (OCH_3_), as anticipated by the MEP analysis shown above.

**Figure 4:**
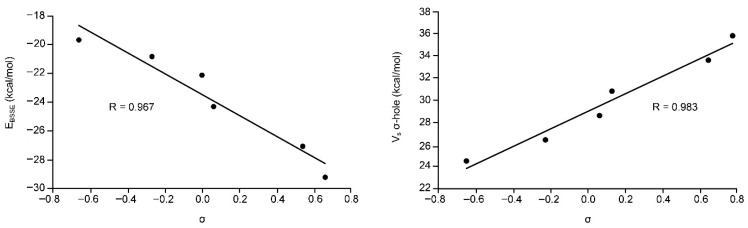
(**Left**) Hammett’s plot (E_BSSE_ vs. σ) of Complexes **7** to **12**. (**Right**) Regression plot of the MEP values at the σ-hole (V_s_ σ-hole) versus the aromatic *p*-substituent constant (σ) for Complexes **7** to **12**.

We have also represented the Hammett’s plot for all complexes used in this study. On the left side of Figure 4 we have plotted the interaction energies (BSSE corrected) vs. the aromatic *p*-substituent constant (σ). As noted, we have obtained a good degree of correlation (r = 0.97). The presence of both electron donating and electron withdrawing groups in the same representation indicates that the influence of the substituents on the binding energies is due to induction effects and that resonance effects are negligible. In addition, we have also represented the MEP values at the σ-hole vs. the aromatic *p*-substituent constant (σ) (Figure 4, right) and obtained a nice correlation (r = 0.98), thus indicating that the influence of the aromatic substituents is mainly attributed to electrostatic effects. This is further confirmed by the slope of both representations that is similar (in absolute value, i.e., 7.3 for the Hammet’s plot and 8.5 for the V_s_ vs. σ_p_ plot).

### 2.3. AIM and NBO Analyses

**Figure 5:**
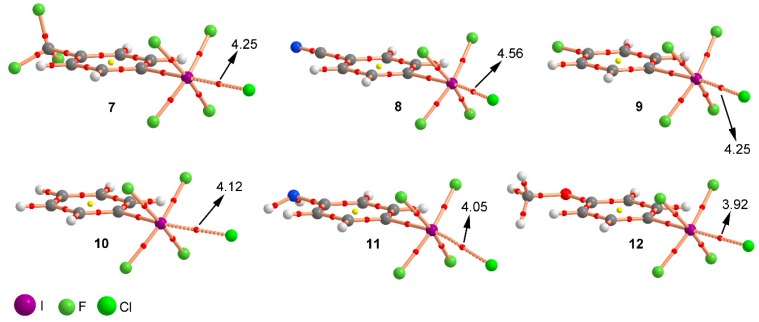
Distribution of critical points (red spheres) and bond paths for Complexes **7** to **12** at the RI-MP2/def2-TZVP level of theory. Bond and ring CPs are represented by red and yellow spheres, respectively. The values of the charge density (10^2^ x ρ) at the bond critical points that emerge upon complexation are indicated in a.u.

We have used the Bader’s AIM theory [45] to characterize the noncovalent interactions present in Complexes **7**–**12**. A bond critical point (CP) and a bond path connecting two atoms is an unambiguous evidence of interaction. The AIM distribution of critical points and bond paths computed for all complexes are shown in Figure 5. As noted, in all cases a bond CP and bond path connect the iodine atom to the Cl^−^, thus characterizing the multivalent halogen bonding interaction. In addition, the value of the Laplacian in all cases is positive, as it is common in closed shell calculations.

In order to study if orbital contributions are important to explain the halogen bond complexes described above, we have performed NBO calculations focusing our attention on the second order perturbation analysis, due to its usefulness in the analysis of donor–acceptor interactions [46]. The results are summarized in Table 2 and in all cases a common behavior is observed, that is, the main orbital contribution comes from the interaction between the lone pairs (LP) of the Cl^−^ to the antibonding (BD*) C–I orbital. In addition, the value of the orbital interactions involving electron withdrawing substituents (Complexes **7** and **8**) is larger than for the rest of the set, in agreement with the interaction energy values obtained. Finally, it is also worth pointing out that the magnitude of the orbital contributions is remarkable compared to the total interaction energy (~70% for Complex **8** and ~80% for Complexes **9** and **10**), thus remarking their importance on the global stability of the complexes studied.

### 2.4. CSD Search

**Figure 6:**
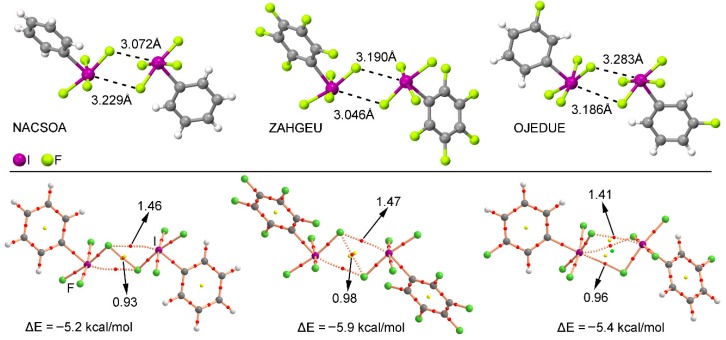
(**Top**) Partial views of the X-ray structure of some multivalent iodine derivatives establishing halogen bonding interactions. The CSD codes are indicated. (**Bottom**) AIM analysis of NACSOA, ZAHGEU, and OJEDUE structures at the MP2/def2-TZVP level of theory. Bond and ring CPs are represented by red, yellow, and green spheres, respectively. The values of the charge density (10^2^ x ρ) at the bond critical points that emerge upon complexation are indicated in a.u.

We have explored the CSD (version 5.38) to find evidence of the importance of multivalent halogen bonding interactions in the crystal structures of iodine derivatives. We have restricted our search to only organic iodine derivatives and found five structures (see Supplementary material for the complete list), three of those containing a substituted aromatic ring. These examples are shown in the top section of Figure 6. As noted, in all three structures—NACSOA [47], ZAHGEU [48], and OJEDUE [49]—the formation of self-assembled dimers involving two discrete units dominates the crystal packing, thus giving relevance to the multivalent halogen bonding interactions in solid state chemistry. In order to shed some light on the energetic stability of these dimers, we have optimized them and in all cases attractive and moderately strong interaction energy values were obtained, ranging between −5.9 and −5.2 kcal/mol, thus remarking the importance of multivalent halogen bonding interactions as a predominant driving force in the self-assembly process. More in particular, complexes involving ZAHGEU structure obtained the largest interaction energy value of all three, since both aromatic moieties are perfluorinated. On the other hand, among complexes involving NACSOA and OJEDUE structures, the latter achieved a larger binding energy value (−5.4 kcal/mol). Moreover, the distances obtained are also within the range of the ones observed in the solid state, giving reliability to the results derived from the RI-MP2 method as well as confirming the importance of these interactions in the crystal structure of multivalent iodine compounds.

In order to further analyze and describe the multivalent halogen bonding interactions observed in these three structures, we have performed an AIM (atoms in molecules) analysis also shown in Figure 6 at the bottom. As noted, in NACSOA and ZAHGEU two symmetrically distributed bond CPs connect the fluorine and iodine atoms of both units, thus characterizing the multivalent halogen bonding interactions. In addition, a bond CP connecting the fluorine atoms of both moieties can also be noted, indicating the presence of F···F contacts. On the other hand, in OJEDUE two symmetrically distributed bond CPs connect two fluorine atoms of one moiety and the iodine atom present in the other, thus indicating a bifurcated multivalent halogen bonding interaction. In addition, a single bond CP and bond path interconnect the fluorine and iodine atoms, similarly to NACSOA and ZAHGEU structures. Moreover, in all three structures two ring CPs emerge upon complexation due to the formation of two supramolecular rings. Finally, in OJEDUE, a cage critical point is also present due to the formation of a supramolecular cage. The values of the Laplacian are in all cases positive, as in common for closed shell calculations.

We have also performed the NBO analysis to evaluate the orbital contributions present in NACSAO, ZAHGEU, and OJEDUE structures by means of the second order perturbation analysis. The results are gathered in Table 3. In all cases, the orbital contribution involves the interaction between a lone pair (LP) of the fluorine atom and the antibonding (BD*) C–I orbital, in agreement with the AIM analysis shown above. In addition, in OJEDUE structure, the orbital contribution is more relevant (0.76 kcal/mol) than in NACSOA and ZAGHEU (0.56 and 0.54 kcal/mol, respectively). Finally, the magnitude of the orbital interactions for all three structures is modest compared to the total interaction energy (~10% for NACSOA and ZAGHEU and ~15% for OJEDUE).

## 3. Theoretical Methods

The geometries of the complexes studied herein have been fully optimized at the RI-MP2/def2-TZVPD level of theory (see Supplementary Material for Cartesian coordinates). For iodine, pseudopotentials were used to accelerate the calculations and to account for relativistic effects, which cannot be neglected. The calculations have been performed by using the program TURBOMOLE version 7.0 [50] and the interaction energy values were obtained using the formula E_int_ = E_AB_ − E_A_ − E_B_ where E_AB_ corresponds to the energy of the optimized complex while E_A_ and E_B_ refer to the energies of the optimized isolated monomers. The *C*_s_ symmetry point group has been used in the optimization of the complexes. The interaction energies were calculated with correction for the basis set superposition error (BSSE) by using the Boys–Bernardi counterpoise [51]. The NBO analysis has been carried out at the HF/def2-TZVP level of theory. The Bader’s “atoms in molecules” theory has been used to study the interactions discussed herein by means of the AIMAll calculation package [52]. The calculations for the wave function and NBO analyses have been performed by means of the Gaussian 09 calculation package [53]. This level of theory has been successfully used before to study similar systems and interactions [54,55].

## 4. Conclusions

In conclusion, we have demonstrated the importance of substituent effects (aromatic substitution) in a series of multivalent halogen bonding complexes. We have used C_6_H_4_(IF_4_)Y (Y = CF_3_, CN, F, H, NH_2_, and OCH_3_) as σ-hole bond donors and Cl^−^ as electron rich entity. In particular, we have used the Hammett’s plot to analyze the influence of the aromatic substitution on the binding energies and we have evidenced an important correlation between the interaction energy values (E_BSSE_) and the *p*-substituted σ values. Besides, we demonstrated the direct correlation between electrostatic effects (MEP values) and the Hammett’s σ parameter. Furthermore, we have also used AIM and NBO computational tools to further characterize the interactions described above. More in particular, orbital interactions involving the lone pairs of Cl^−^ and the antibonding (BD*) C–I orbital are an important source of stability of the complexes studied. Finally, several experimental examples retrieved from the CSD give reliability to the results derived from calculations and highlight the importance of these interactions in the solid state of multivalent iodine compounds.

## Figures and Tables

**Table 1:** Interaction energies without and with BSSE correction (ΔE and ΔE_BSSE_ respectively, kcal/mol), equilibrium distances (R, Å), and value of the density at the bond CP (10^2^ x ρ, a.u.) for Complexes **7**–**12** at the RI-MP2/def2-TZVPD level of theory.

Complex	ΔE	ΔE_BSSE_	R	10^2^ x ρ
**7**	−28.8	−27.1	2.837	4.25
**8**	−31.2	−29.2	2.821	4.56
**9**	−26.2	−24.3	2.783	4.25
**10**	−24.0	−22.1	2.846	4.12
**11**	−21.5	−19.7	2.826	4.05
**12**	−22.5	−20.8	2.867	3.92

**Table 2:** Donor and acceptor NBOs with indication of the second-order interaction energy E^(2)^ (kcal/mol) for Complexes **7**–**12**.

Complex	Donor ^a^	Acceptor	E^(2)^
**7**	LP Cl	BD* C–I	20.7
**8**	LP Cl	BD* C–I	21.0
**9**	LP Cl	BD* C–I	18.5
**10**	LP Cl	BD* C–I	17.5
**11**	LP Cl	BD* C–I	16.9
**12**	LP Cl	BD* C–I	17.3

^a^ LP, BD* stand for lone pair and anti-bonding orbital, respectively.

**Table 3:** Donor and acceptor NBOs with indication of the second-order interaction energy E^(2)^ (kcal/mol) for NACSOA, ZAGHEU, and OJEDUE.

CCDC Code	Donor ^a^	Acceptor	E^(2)^
**NACSOA**	LP F	BD* C–I	0.56
**ZAGHEU**	LP F	BD* C–I	0.54
**OJEDUE**	LP F	BD* C–I	0.76

^a^ LP, BD* stand for lone pair and anti-bonding orbital, respectively.

## Supplementary Materials

Supplementary materials are available on line. Cartesian coordinates of optimized complexes and the list of X-ray structures obtained from the CSD search.

## Abbreviations

The following abbreviations are used in this manuscript:

DOAJ	Directory of open access journals
AIM	Atoms in molecules
IUPAC	International Union of Pure and Applied Chemistry
MEP	Molecular electrostatic potential
MP2	Second order Moller–Plesset
BSSE	Basis Set Superposition Error
CSD	Cambridge Structural Database
CP	Critical point
NBO	Natural Bonding Orbital
MINECO	Ministerio de Economía y Competitividad
