# Supplementary material for: Substituent Effects in Multivalent Halogen Bonding Complexes: A Combined Theoretical and Crystallographic Study
Source: Molecules. 2017 Dec 22;23(1):18. doi: 10.3390/molecules23010018 (PMC5943962; doi:10.3390/molecules23010018)
Supplement: Supplementary file 1 [file molecules-23-00018-s001.pdf]

Substituent effects in multivalent halogen bonding complexes: A combined theoretical and crystallographic study.

Antonio Bauzá,[1*] and Antonio Frontera,[1]*

[1]Department of Chemistry Universitat de les Illes Balears, Crta. de Valldemossa km 7.5, 07122 Palma (Baleares), Spain. E-mail: antonio.bauza@uib.es, toni.frontera@uib.es;

Fax: +34 971 173426

Electronic Supplementary Information

Cartesian Coordinates

7.

I	3.0035670	0.0091954	0.0000000
F	2.7453897	1.3756055	1.3907544
F	2.7453897	1.3756055	-1.3907544
F	2.7621133	-1.3610026	-1.3889896
F	2.7621133	-1.3610026	1.3889896
C	0.8892698	-0.0030725	0.0000000
C	0.2106063	-0.0085844	1.2144038
C	0.2106063	-0.0085844	-1.2144038
C	-1.1808967	-0.0201719	1.2117179
H	0.7626986	-0.0064865	2.1458055
C	-1.1808967	-0.0201719	-1.2117179
H	0.7626986	-0.0064865	-2.1458055
C	-1.8716341	-0.0301736	0.0000000
H	-1.7273550	-0.0292007	2.1480697
H	-1.7273550	-0.0292007	-2.1480697
Cl	5.8266311	0.0149757	0.0000000
C	-3.3646035	0.0109048	0.0000000

F	-3.8955188	-0.5896738	-1.0813958
F	-3.8373051	1.2771989	0.0000000
F	-3.8955188	-0.5896738	1.0813958

8.

I	2.6142916	-0.1287115	0.0000000
F	2.3976116	1.2545964	-1.3382580
F	2.2912971	-1.4972184	-1.4192163
F	2.2912971	-1.4972184	1.4192163
F	2.3976116	1.2545964	1.3382580
C	0.5012142	-0.0544346	0.0000000
C	-0.1426955	1.1797995	0.0000000
C	-0.2123516	-1.2495552	0.0000000
C	-1.5322339	1.2203817	0.0000000
H	0.4359868	2.0949711	0.0000000
C	-1.6017027	-1.2105691	0.0000000
H	0.3149179	-2.1952246	0.0000000
C	-2.2623358	0.0248260	0.0000000
H	-2.0572138	2.1695131	0.0000000
H	-2.1803145	-2.1279796	0.0000000
Cl	5.3010079	0.5976557	0.0000000
C	-3.6912832	0.0656173	0.0000000
N	-4.8651048	0.0989543	0.0000000

9.

I	2.3099139	-0.1213436	0.0000000
F	2.1115824	1.2656816	-1.3416360
F	2.0039421	-1.4959765	-1.4105754
F	2.0039421	-1.4959765	1.4105754

F	2.1115824	1.2656816	1.3416360
C	0.2060961	-0.0452912	0.0000000
C	-0.4351563	1.1889133	0.0000000
C	-0.5106241	-1.2369940	0.0000000
C	-1.8278659	1.2331409	0.0000000
H	0.1433658	2.1039445	0.0000000
C	-1.9031772	-1.1946803	0.0000000
H	0.0114508	-2.1853264	0.0000000
C	-2.5311113	0.0399213	0.0000000
H	-2.3653159	2.1744499	0.0000000
H	-2.4981326	-2.1007324	0.0000000
Cl	5.0564441	0.5225805	0.0000000
F	-3.8869364	0.0820073	0.0000000

10.

I	2.2922183	0.1181900	0.0000000
F	1.9916880	1.4931368	-1.4090297
F	2.0972000	-1.2686335	-1.3438116
F	2.0972000	-1.2686335	1.3438116
F	1.9916880	1.4931368	1.4090297
C	0.1866245	0.0437142	0.0000000
C	-0.5278042	1.2370238	0.0000000
C	-0.4525930	-1.1917698	0.0000000
C	-1.9204697	1.1852745	0.0000000
H	-0.0029177	2.1839354	0.0000000
C	-1.8459802	-1.2262162	0.0000000
H	0.1284568	-2.1053425	0.0000000
C	-2.5820260	-0.0421075	0.0000000
H	-2.4902293	2.1090765	0.0000000

H	-2.3573231	-2.1835935	0.0000000
H	-3.6667191	-0.0756544	0.0000000
Cl	5.0609865	-0.5015371	0.0000000

11.

I	2.7707496	-0.1577831	0.0000000
F	2.5702319	1.2161277	1.3551801
F	2.5702319	1.2161277	-1.3551801
F	2.4921428	-1.5012329	-1.4462842
F	2.4921428	-1.5012329	1.4462842
C	0.6732400	-0.1042427	0.0000000
C	-0.0114755	-0.0828472	1.2094964
C	-0.0114755	-0.0828472	-1.2094964
C	-1.4017959	-0.0342751	1.2022985
H	0.5335168	-0.1046375	2.1447814
C	-1.4017959	-0.0342751	-1.2022985
H	0.5335168	-0.1046375	-2.1447814
C	-2.1120399	-0.0020513	0.0000000
H	-1.9437148	-0.0207338	2.1446045
H	-1.9437148	-0.0207338	-2.1446045
Cl	5.5430057	0.4873995	0.0000000
N	-3.5202359	-0.0054898	0.0000000
H	-3.9162649	0.4186824	0.8293611
H	-3.9162649	0.4186824	-0.8293611

12.

I	0.0902637	-3.2262056	0.0000000
F	-1.2894775	-2.9233342	1.3675626
F	1.4917997	-3.0619528	1.3686546

F	1.4917997	-3.0619528	-1.3686546
F	-1.2894775	-2.9233342	-1.3675626
C	0.1978419	-1.1291558	0.0000000
C	-0.9738717	-0.3880668	0.0000000
C	1.4429807	-0.5047128	0.0000000
C	-0.9097867	1.0068144	0.0000000
H	-1.9346987	-0.8876056	0.0000000
C	1.5045571	0.8818275	0.0000000
H	2.3499293	-1.0962157	0.0000000
C	0.3324236	1.6412619	0.0000000
H	-1.8325593	1.5728554	0.0000000
H	2.4581039	1.3993143	0.0000000
Cl	-0.0601027	-6.0897313	0.0000000
O	0.5061667	3.0015002	0.0000000
C	-0.6737544	3.7829133	0.0000000
H	-0.3455679	4.8206387	0.0000000
H	-1.2782850	3.5925709	-0.8924239
H	-1.2782850	3.5925709	0.8924239

CCDC Codes

NACSOA, OJEDUE, ZAHFUJ, ZAHGEU, ZAZJOB
